# The relationship between levels of interpersonal sensitivity and help-seeking behavior in individuals with substance use disorders: A cross-sectional study

**DOI:** 10.1097/MD.0000000000048789

**Published:** 2026-05-22

**Authors:** Zelal Arslan, Dilek Ayakdaş Dağli

**Affiliations:** a Central District Community Health Center, Kutahya Provincial Health Directorate Kütahya, Kütahya, Türkiye; b Department of Mental Health and Psychiatry Nursing, Izmir Katip Celebi University, Faculty of Health Science, Izmir, Türkiye.

**Keywords:** help-seeking behavior, interpersonal sensitivity, substance use disorder

## Abstract

This study aims to examine the effect of interpersonal sensitivity (IPSM) levels in individuals with substance use disorders on their help-seeking behavior. The study is a descriptive, cross-sectional, and correlational quantitative research. Data were collected face-to-face from 115 individuals undergoing inpatient treatment at the Alcohol and Substance Treatment Centre. Data collection tools included a Demographic Information Form, the Interpersonal Sensitivity Scale, and the Help-Seeking Attitude Scale. Descriptive statistical analyses were used to evaluate the data, while correlation analysis and linear regression analysis were used to evaluate the relationship between the scales. The mean age of the patients was 35.57 ± 11.43, and 64.3% had previously undergone treatment. When examining the relationship between the scales, a statistically significant relationship was found between the mean scores of IPSM and interpersonal openness (*R* = 0.408), neediness (*R* = 0.208), and social acceptance (*R* = 0.294) subscales of the help-seeking attitude. Consequently, a significant relationship has been found between the total mean scores on the IPS scale and the total mean scores on the help-seeking behavior subscales among individuals with substance use disorders. Accordingly, as individuals’ level of IPS increases, their perceptions of social acceptance and their need to seek help decrease.

## 1. Introduction

Substance use disorders (SUDs) have become a significant public health issue worldwide and in Turkey, negatively impacting the health of individuals and society.^[1]^ The World Health Organization defines SUD as “a brain disease characterized by a strong craving for a substance (such as alcohol, tobacco, cannabis, opioids, stimulants, etc), loss of control, development of tolerance to the substance, and withdrawal symptoms when not using it, resulting from repeated use of the substance.”^[2]^ Alcohol and substance-related deaths are increasing in Turkey and around the World.^[3]^ In this regard, addiction treatment is important. Addiction is a chronic disease characterized by relapse and remission.^[4,5]^ Relapse is a situation in which an individual who has not used alcohol/substances for a long time ends up using alcohol/substances again.^[6]^ Among the reasons for starting substance use and relapse, individuals experience interpersonal conflict, enter a repetitive cycle, lose hope for recovery, experience anxiety, feel negative emotions and thoughts, and experience internalized stigma.^[5,7]^ The negative feelings and thoughts experienced by individuals lead to low self-esteem and impaired interpersonal relationships. These problems cause individuals to experience hopelessness, feelings of inadequacy, intense stress, difficulty establishing interpersonal relationships, and isolation due to feelings of being misunderstood. As a result of these difficulties, they do not seek help and try to cope with negative situations by using substances.^[8]^ The high level of interpersonal sensitivity (IPS) of individuals with SUD may be the cause of these negative situations. IPS is defined as the individual’s perception, interpretation, and understanding of ideas developed about the outside world and others.^[9]^ Individuals with high IPS have an unusual sensitivity to the ideas of others. This sensitivity causes the individual to be sensitive to rejection. Individuals may exhibit social withdrawal to avoid rejection. Individuals with high IPS experience problems in their relationships, and these problems can also negatively affect the individual’s emotional state. In a study conducted by Otani, it was noted that individuals with high IPS struggle with self-management and exhibit avoidance behavior in the face of negative situations.^[10]^ Other studies on the concept of IPS have also found that individuals with high levels of IPS experience social withdrawal, anxiety, and mood disorders, and use self-medication with alcohol or drugs as a coping strategy.^[11–13]^ At the same time, it has been observed that individuals with high IPS have high feelings of inadequacy, accompanied by low self-esteem and fears of rejection.^[9,14,15]^ Intense feelings of inadequacy and low self-esteem cause social adjustment disorders in individuals.^[16]^ It is thought that these problems experienced by individuals in their interpersonal relationships will also affect their help-seeking behavior. Help-seeking behavior is defined as the process of disclosing one’s situation to another person when the individual is unable to cope with the crisis they are facing.^[17]^ Help-seeking behavior is one of the components necessary for individuals with SUD to achieve recovery.^[18]^ The help-seeking behavior of individuals diagnosed with SUD may be influenced not only by individual factors but also by cultural and social factors.^[19]^ One environmental factor, “stigmatization,” limits individuals’ help-seeking behavior. Stigmatization is characterized by the negative perceptions and stereotypes held by the majority towards a particular group.^[20,21]^ When individuals internalize these stereotypes, they do not seek treatment, and it affects their self-esteem, preventing them from seeking support.^[22]^ Studies have shown that individuals diagnosed with SUD are more affected by the illness than individuals with other psychiatric diagnoses, yet they seek treatment less frequently.^[23–25]^ The reasons for not seeking help include lack of social support, negative feelings and thoughts, and internalized stigma.^[26]^ These negative feelings and thoughts experienced by individuals affect their help-seeking behavior, while high IPS can increase the severity of these symptoms.^[27]^ This can emerge as a significant barrier affecting an individual’s help-seeking behavior. Based on this premise, determining the relationship between IPS levels and help-seeking behavior in individuals with SUD may be important for preventing the onset of substance use and relapses, as well as facilitating recovery. A review of the literature reveals no studies evaluating IPS and help-seeking behavior in individuals with SUD. The aim of this study is to examine the relationship between IPS levels and help-seeking behavior in individuals with SUD.

### 1.1. Research question

What is the level of IPS in individuals with SUDs?

What is the level of help-seeking behavior in individuals with SUDs?

Is there a relationship between interpersonal sensitivity measure (IPSM) levels and help-seeking behavior in individuals with SUDs?

### 1.2. Hypothesis

H1: There is a relationship between IPSM levels and help-seeking behavior in individuals diagnosed with SUD.

## 2. Materials and methods

### 2.1. Type of research

The study is a cross-sectional and correlational investigation examining the effect of IPSM levels on help-seeking behavior among individuals diagnosed with SUD.

### 2.2. Participants

The population of the study consisted of 140 patients with SUD who were hospitalized in the Alcohol and Substance Addiction Treatment Center service of a regional psychiatric hospital between March and December 2024. Data were collected face-to-face from 115 individuals who met the inclusion criteria. The inclusion criteria for the study were defined as individuals who were 18 years of age or older, receiving inpatient treatment at the clinic, diagnosed with SUD, had completed the detoxification phase, and did not have any psychotic disorder.

### 2.3. Instruments

The data for the study were collected using 3 measurement instruments.

#### 2.3.1. Demographic Information Form

This consists of a total of 23 questions, including 7 questions covering the sociodemographic data of patients diagnosed with SUD and 16 questions covering their substance use history.

#### 2.3.2. Help-Seeking Attitude Scale

The scale was developed by Özbay and colleagues to examine attitudes toward seeking professional psychological help.^[28]^ The scale consists of 32 items and has 5 subscales (“Interpersonal openness,” “Distress,” “Belief in counseling,” “Feeling of need,” and “Social acceptance”). No total score is obtained from the scale. Each subscale is evaluated separately.

(1) Interpersonal openness: includes topics such as self-disclosure at a personal level that may influence an individual’s decision to seek help or not, sharing private information with others, social shyness, social rigidity, introversion, and a tendency toward anonymity. As interpersonal openness scores increase, individuals’ interpersonal openness decreases.
(2) Distress; this is a dimension aimed at determining whether tendencies to seek help emerge when pathology increases. In the scale evaluation, it was noted that as the Stress Area score increased, the individual did not experience distress in seeking help.
(3) Belief in counseling: this encompasses belief and trust in professional assistance services and individuals. In the scale evaluation, as the area of belief in counseling scores increase, the individual’s belief in seeking help also increases.
(4) Feeling of need: interpreted as the individual’s perception and acceptance of their psychological problem. In the scale evaluation, it has been stated that as the mean scores in the feeling of need area increase, the individual does not feel the need to seek help.
(5) Social acceptance: this refers to negative attitudes and social cognitions regarding psychological help. In the scale evaluation, as the Social Acceptance Area scores increase, the individual’s belief in social acceptance decreases ^[28]^.

#### 2.3.3. IPSM

The scale examines individuals’ levels of IPS. It was developed by Boyce and Parker,^[9]^ and its validity and reliability in Turkish were established by Doğan and Sapmaz.^[29]^ The scale consists of 30 items and 3 subscales (interpersonal anxiety and dependency, lack of social self-confidence, and nonassertive behaviors). The scale ranges from a minimum score of 30 to a maximum score of 150. A higher score on the scale indicates an increase in the individual’s level of IPS. According to the results of the reliability analyses, the Cronbach alpha internal consistency coefficient for the scale as a whole is 0.81. In this study, the Cronbach alpha value is 0.82.

### 2.4. Statistical analysis

The data obtained in the study were analyzed using the free trial version of Statistical Package for Social Sciences Statistics (SPSS; IBM SPSS Statistics 25.0) for Windows 25.0. Descriptive statistical methods (number, percentage, mean, and standard deviation) were used to evaluate the data. The normality of the data used depends on the skewness and kurtosis values being between ± 2. The variables were found to be normally distributed. The relationship between the variables was analyzed using Pearson correlation analysis. A “reliability analysis” was applied to test the reliability of the scales. The effect of independent variables on the dependent variable was examined using multiple linear regression analysis. In the study, *P*-values below .05 were considered significant.

### 2.5. Ethical considerations

For the implementation of the study, ethical approval was obtained from the Non-Interventional Research Ethics Committee of a university with the date January 18, 2024 and number 0029, and permission was obtained from the administration of the institution where the research would be conducted. In addition, verbal and written informed consent was obtained from the patients participating in the study. This study adhered to the principles outlined in the Declaration of Helsinki.

## 3. Results

### 3.1. Participant characteristics

It was found that all SUD individuals were male, with an average age of 35.57 ± 11.43. It was observed that 43.5% of individuals were single, 56.5% had an income equal to their expenses, 34.8% lived in a metropolitan city, 42.6% had a high school education, and the majority were employed (Table 1).

**Table 1:** Distribution of individuals with substance use disorder according to descriptive characteristics.

		Number	%
Yaş: 35.57 ± 11.43			
Gender	Female	0	0.0
	Male	115	100.0
Marital status	Single	50	43.5
	Married	44	38.3
	Divorced	21	18.2
Income status	Income less than expenditure	22	19.1
	Income equal to expenditure	65	56.5
	Income greater than expenditure	28	24.3
Longest place of residence	Village-Town	20	17.4
	District	30	26.1
	City	25	21.7
	Metropolitan city	40	34.8
Educational status	Literate	2	1.7
	Primary school graduate	54	47.0
	High school graduate	49	42.6
	University graduate	10	8.7
Job	Civil servant	4	3.5
	Worker	50	43.4
	Retired	9	7.8
	Tradesman	29	25.2
	Unemployed, seeking work	11	9.6
	Other	12	10.4
Total		115	100

When examining the distribution of alcohol and substance use histories among individuals with SUD, it was observed that the age of onset of substance use was 19.04 ± 6.78. It was reported that 78.3% of individuals used alcohol, 61.7% used substances, and 47.0% started using alcohol and substances due to peer influence. It was determined that 64.3% of individuals had previously undergone treatment and 63.5% sought treatment voluntarily (Table 2).

**Table 2:** Distribution of individuals with substance use disorders according to alcohol and substance use characteristics.

		Number	%
Age of onset of alcohol/substance use: 19.04 ± 6.78			
Alcohol use	No	25	21.7
	Yes	90	78.3
Substance use	No	44	38.3
	Yes	71	61.7
Substance used*	Marijuana	43	37.4
	Heroin	5	4.3
	Cocaine	25	21.7
	Synthetic drugs	21	18.3
	Ecstasy	30	26.1
	Amphetamine	6	5.2
	Methamphetamine	45	39.1
	Multiple substances	26	22.6
How alcohol and substance use began	Curiosity	21	18.3
	Escaping negative emotions	26	22.6
	Influence of peers	54	47.0
	Military service	1	0.9
	Other (environmental factors)	13	11.3
Alcohol use in the family	Yes	41	35.7
	No	74	64.3
Previous treatment status	Yes	74	64.3
	No	41	35.7
Reason for resuming alcohol and substance use	Conflict (work/family)	18	15.7
	Escaping negative emotions	27	23.5
	Influence of environment	47	40.9
	Lack of social support	4	3.5
	Other (inability to fulfill roles, trauma, and stigmatization)	19	16.5
Reason for starting treatment	Family pressure	1	0.9
	My own choice	73	63.5
	Both	41	35.7
Total		115	100

* Has been multiplied.

### 3.2. IPSM and Help-Seeking Attitude Scale Scores

In the study, the mean total scores for the subdimensions of the Help-Seeking Attitude Scale were, respectively, interpersonal openness 40.10 ± 10.81, distress 26.75 ± 6.82, belief in counseling 31.20 ± 6.37, feeling of need 17.21 ± 5.16, and social acceptance 4.26 ± 3.15. The total mean score for the IPSM was 86.60 ± 15.79, and the subscale total mean scores were 48.46 ± 11.53 for interpersonal anxiety and dependency, 15.74 ± 4.97 for social self-confidence deficiency, and nonassertive behavior 22.40 ± 5.55 (Table 3).

**Table 3:** Distribution of mean scores on the interpersonal sensitivity measure and Help-Seeking Attitude Scale for individuals with substance use disorders.

	Scales	Min	Max	$\bar{x}$	SS	Min	Max
Help-Seeking Attitude Scale	Interpersonal openness	19	63	40.10	10.81	12	72
	Distress	11	36	26.75	6.82	6	36
	Belief in counseling	13	42	31.20	6.37	7	42
	Feeling of need	5	30	17.21	5.16	5	30
	Social acceptance	2	12	4.26	3.15	2	12
Interpersonal sensitivity measure	Interpersonal anxiety and dependence	18	78	48.46	11.53	16	80
	Lack of social self-confidence	7	33	15.74	4.97	7	35
	Nonassertive behavior	7	35	22.40	5.55	7	35
	Interpersonal Sensitivity Measure (IPSM)	46	127	86.60	15.79	30	150

### 3.3. The relationship between IPSM and help-seeking attitudes among individuals with SUDs

There was a statistically significant and positive correlation between the interpersonal openness mean score and the interpersonal anxiety and dependency mean score (*R* = 0.401, *P* < .01), a statistically significant and positively directional relationship (*R* = 0.259, *P* < .01) between the mean interpersonal openness score and the mean nonassertive behavior score, and a statistically significant and positively directional relationship (*R* = 0.408, *P* < .01) between the mean interpersonal openness score and the IPSM. A statistically significant and negative correlation (*r* = −0.197, *P* < .05) was found between the average belief in counseling and the average lack of social self-confidence deficiency score, a statistically significant and positive relationship (*R* = 0.186, *P* < .05) was found between the feeling of need score average and the nonassertive behaviors score average, and a statistically significant and positive relationship (*R* = 0.208, *P* < .05) was found between the feeling of need score average and IPSM. There was a statistically significant and positive correlation between the average social acceptance score and the average interpersonal anxiety and dependency scores (*R* = 0.249, *P* < .01). A statistically significant and positively directional relationship (*R* = 0.238, *P* < .05) was found between the average social acceptance score and the average lack of social self-confidence score, and a statistically significant and positively directional relationship (*R* = 0.294, *P* < .01) was found between the average social acceptance score and IPSM (Table 4).

**Table 4:** The relationship between interpersonal sensitivity and help-seeking attitudes among individuals with substance use disorders.

		Help-Seeking Attitude Scale				
		Interpersonal openness	Distress	Belief in counseling	Feeling of need	Social acceptance
Interpersonal Sensitivity Measure	Interpersonal anxiety and dependency	0.401**	0.085	0.101	0.181	0.249**
	Lack of social self-confidence	0.077	−0.106	−0.197*	0.033	0.238*
	Nonassertive behavior	0.259**	−0.110	0.008	0.186*	0.106
	Interpersonal Sensitivity Measure	0.408**	−0.010	0.015	0.208*	0.294**

* *P* < .05, Pearson correlation.

** *P* < .01, Pearson correlation.

### 3.4. The effect of IPSM levels of individuals with SUDs on their help-seeking attitudes

IPSM, have a statistically significant effect on the interpersonal openness subdimension of the help-seeking attitude scale (*P* < .05). It is seen that 14.8% of the variation in interpersonal openness is explained by the independent variables (adjusted *R*^2^ = 0.148).

The interpersonal anxiety and dependence subdimension of the IPSM and the social self-confidence deficiency subdimension were found to have a significant effect on the social acceptance subdimension of the help-seeking attitude scale (*P* < .05). It is observed that 9.1% of the variation in social acceptance is explained by independent variables (adjusted *R*^2^ = 0.091) (Table 5).

**Table 5:** The effect of interpersonal sensitivity levels of individuals with substance use disorders on their help-seeking attitudes.

Dependent variable	Dependent variable	ß	t	*P*	Beta	VIF	*F*	*P*	*R* ^2^	Durbin Watson%
Interpersonal openness	Constant	17.443	3.126	0.002*			7.619	0.000	0.148	1.734
	Interpersonal anxiety and dependency	0.330	3.486	0.001*	0.352	1.363				
	Lack of social confidence	0.169	0.886	0.378	0.078	1.033				
	Nonassertive behavior	0.179	0.901	0.370	0.092	1.387				
Social acceptance	Constant	−1.392	−0.828	0.409			4.813	0.003	0.091	2.069
	Interpersonal anxiety and dependency	0.063	2.209	0.029*	0.230	1.363				
	Lack of social confidence	0.148	2.573	0.011*	0.233	1.033				
	nonassertive behavior	0.012	0.203	0.839	0.021	1.387				

ß = unstandardized coefficient, Beta (β) = standardized coefficient, VIF = variance inflation factor, R^2^ = determination coefficient.

* *P* < .05.

## 4. Discussion

This study, conducted to examine the effect of IPSM levels on help-seeking behavior in individuals with SUD, found that individuals with SUD were predominantly male, with an average age of 35.57 ± 11.43, mostly single, primary school graduates, and employed. The research findings are consistent with the literature.^[30–32]^

The study found that the IPS levels of the individuals were 86.60 ± 15.79, which is above average. In a study conducted by Baysan Arabaci and colleagues on student nurses, they stated that students with high levels of IPS had high social media addiction.^[33]^ In their study, Hall and Neighbors found that IPSM mediated alcohol use.^[13]^ IPSM is a state in which an individual shows excessive sensitivity to the feelings, thoughts, and behaviors of others and intensely perceives and interprets the evaluations of others in social environments.^[34]^ Research indicates that reasons for relapse into alcohol/substance use among individuals with SUD include experiencing interpersonal conflict and escaping negative emotions.^[35–37]^ Indeed, in this study, when the reasons for starting substance use and the reasons for relapse in individuals were examined, it was seen that individuals experienced interpersonal conflicts and had a desire to escape negative emotions. Excessive sensitivity in individuals with high IPS levels can lead to interpersonal conflict. Furthermore, intense perception levels of evaluations can also cause negative emotions.^[11]^ When these results are evaluated holistically, it can be said that the IPS level of individuals with SUD is a risk factor for starting substance use and relapse. In the study, the mean scores for interpersonal anxiety and dependency, which are subdimensions of IPS, were at the average level, while nonassertive behaviors were above the average level. Nonassertive behaviors are explained as avoidance of social relationships and environments due to excessive anxiety experienced by individuals.^[29]^ Individuals with SUD may exhibit nonassertive behaviors and withdraw from the environment due to the intense stress they experience and the belief that they will not be understood. Individuals may turn to substance use to cope with this situation they are experiencing. Studies report that individuals who avoid interpersonal relationships and whose social functioning is negatively affected face many difficulties.^[38,39]^ When faced with a stressful situation, individuals may resort to substance use, which is a coping skill they have previously experienced, and this can lead to relapse.^[40]^ Indeed, 64.3% of individuals had previously undergone treatment, and 39.2% stated that the reason for restarting treatment was to avoid interpersonal conflicts and escape negative emotions.

In the study, when the subscales of the Help-Seeking Attitude Scale were examined, individuals’ interpersonal openness and social acceptance subscale mean scores were below average, while their “distress” and “belief in counseling” subscale mean scores were above average. In other words, as individuals’ average scores for interpersonal openness and social acceptance decreased, it was clear that they were more accepted by society and more open in their relationships; conversely, as their average scores for distress and belief in counseling increased, it was clear that they did not experience difficulties in seeking help and placed great importance on consulting a professional. Considering that individuals in the study came to the hospital for treatment of their own volition, this is an expected result. Furthermore, the study indicated that individuals decided to undergo treatment of their own accord.

The study found a statistically significant relationship between the interpersonal anxiety and dependency scores of individuals with SUD and the interpersonal openness and social acceptance subdimensions of help-seeking behavior. Individuals with high interpersonal anxiety and dependency experience anxiety about opening up and exhibit reduced help-seeking behavior due to concerns about not being socially accepted. Previous studies have also shown that individuals who avoid interpersonal relationships exhibit a decrease in help-seeking attitudes.^[41,42]^ Indeed, regression analysis revealed that the most important variable affecting the help-seeking attitude subscales of interpersonal openness and social acceptance was interpersonal anxiety and dependency.

There is a statistically significant relationship between the lack of social self-confidence individuals with SUD and the subscales of help-seeking behavior, belief in counseling and social acceptance. The lack of social self-confidence that develops in individuals also affects their belief in counseling. Belief in counseling involves the individual accepting their own mental health problem as well as seeking help from a professional and trusting these individuals.^[28]^ The lack of social confidence in individuals with SUD may limit these individuals’ participation in social interaction and social support mechanisms, which may negatively affect the individual’s belief in solidarity regarding professional help. Individuals with a lack of social confidence do not want to seek help because they fear stigmatization, in other words, a decrease in social acceptance.

The addiction process often brings with it family conflicts and weakened ties with the social environment. Individuals deprived of social support feel lonely and insecure in social settings.^[43,44]^ Indeed, the subdimension of social acceptance from the Help-Seeking Attitude Scale, the subdimensions of social self-confidence and interpersonal anxiety and dependence from the IPSM, predict addiction at a rate of 9%. Although this rate appears statistically limited, it is a finding that cannot be ignored for individuals with SUDs. Considering that there are multiple factors influencing help-seeking behavior (level of stigmatization, social support systems, insight), it is thought to make a clinically meaningful contribution. Nurses establishing trust-based communication with individuals with SUDs, avoiding stigmatization, can support individuals in expressing themselves, thereby preventing interpersonal anxiety and contributing to social acceptance, which may enhance the effectiveness of care.

When the findings were evaluated holistically, it was observed that individuals with anxiety and dependency traits in their interpersonal relationships were not socially accepted and did not want to open up. Furthermore, it was determined that when patients experience a lack of social self-confidence, they do not seek help because they believe that their trust in counseling and social acceptance will decrease. In the regression analysis, IPS levels affect the interpersonal openness and social acceptance subdimensions of the help-seeking attitude subscale. In this regard, the hypothesis of the study was confirmed.

The fact that the research sample was drawn from a single center and consisted solely of men limits the generalizability of the findings. Gender and culture may be significant variables influencing IPS and help-seeking behavior. Research has identified 3 key conditions for men to seek help: the validation of masculinity norms, self-stigmatization, and attitudes towards help-seeking.^[45,46]^ Especially in patriarchal societies such as Turkish society, culture has assigned men a significant role. Characteristics expected of men by culture – such as being a strong individual, solving problems independently, effectively managing family dynamics, and being self-reliant – may have limited their tendency to seek help and influenced their levels of IPS. Therefore, the findings of this study may shed light on male populations with similar cultural backgrounds. Although our study did not aim to make comparisons, future research could examine how gender and cultural differences influence levels of IPS and help-seeking behavior.

### 4.1. Conclusions and recommendations

Considering that help-seeking behavior is the most important variable in preventing relapses, it may be important to intervene in patients’ IPS traits. Furthermore, interpersonal anxiety and dependency have been found to be the most important variables affecting interpersonal openness, one of the components of help-seeking attitude. In this case, reducing patients’ levels of anxiety and dependency is thought to be important in treatment. Considering the effect of lack of social self-confidence on social acceptance and belief in counseling, it may be important to engage hospitalized patients in activities that will boost their self-esteem.

## Author contributions

**Conceptualization:** Zelal Arslan, Dilek Ayakdaş Dağli.

**Data curation:** Zelal Arslan, Dilek Ayakdaş Dağli.

**Investigation:** Zelal Arslan, Dilek Ayakdaş Dağli.

**Methodology:** Zelal Arslan, Dilek Ayakdaş Dağli.

**Project administration:** Dilek Ayakdaş Dağli.

**Resources:** Zelal Arslan.

**Writing – original draft:** Zelal Arslan.

**Supervision:** Dilek Ayakdaş Dağli.

**Writing – review & editing:** Dilek Ayakdaş Dağli.
